# Phase 1/2 study of valproic acid and short-course radiotherapy plus capecitabine as preoperative treatment in low-moderate risk rectal cancer-V-shoRT-R3 (Valproic acid - short RadioTherapy - rectum 3rd trial)

**DOI:** 10.1186/1471-2407-14-875

**Published:** 2014-11-24

**Authors:** Antonio Avallone, Maria Carmela Piccirillo, Paolo Delrio, Biagio Pecori, Elena Di Gennaro, Luigi Aloj, Fabiana Tatangelo, Valentina D’Angelo, Cinzia Granata, Ernesta Cavalcanti, Nicola Maurea, Piera Maiolino, Franco Bianco, Massimo Montano, Lucrezia Silvestro, Manuela Terranova Barberio, Maria Serena Roca, Massimo Di Maio, Pietro Marone, Gerardo Botti, Antonella Petrillo, Gennaro Daniele, Secondo Lastoria, Vincenzo R Iaffaioli, Giovanni Romano, Corradina Caracò, Paolo Muto, Ciro Gallo, Francesco Perrone, Alfredo Budillon

**Affiliations:** Gastrointestinal Medical Oncology Unit, Istituto Nazionale per lo Studio e la Cura dei Tumori “Fondazione Giovanni Pascale” – IRCCS, Napoli, Italy; Clinical Trials Unit, Istituto Nazionale Tumori “Fondazione G. Pascale” – IRCCS, Via M. Semmola 80131, Napoli, Italy; Colorectal Surgery Unit, Istituto Nazionale per lo Studio e la Cura dei Tumori “Fondazione Giovanni Pascale” – IRCCS, Napoli, Italy; Radiotherapy Unit, Istituto Nazionale per lo Studio e la Cura dei Tumori “Fondazione Giovanni Pascale” – IRCCS, Napoli, Italy; Experimental Pharmacology Unit, Istituto Nazionale per lo Studio e la Cura dei Tumori “Fondazione Giovanni Pascale” – IRCCS, Napoli, Italy; Nuclear Medicine Unit, Istituto Nazionale per lo Studio e la Cura dei Tumori “Fondazione Giovanni Pascale” – IRCCS, Napoli, Italy; Pathology Unit, Istituto Nazionale per lo Studio e la Cura dei Tumori “Fondazione Giovanni Pascale” – IRCCS, Napoli, Italy; Endoscopy Unit, Istituto Nazionale per lo Studio e la Cura dei Tumori “Fondazione Giovanni Pascale” – IRCCS, Napoli, Italy; Radiology Unit, Istituto Nazionale per lo Studio e la Cura dei Tumori “Fondazione Giovanni Pascale” – IRCCS, Napoli, Italy; Clinical Pathology Unit, Istituto Nazionale per lo Studio e la Cura dei Tumori “Fondazione Giovanni Pascale” – IRCCS, Napoli, Italy; Cardiology Unit, Istituto Nazionale per lo Studio e la Cura dei Tumori “Fondazione Giovanni Pascale” – IRCCS, Napoli, Italy; Pharmacy Unit, Istituto Nazionale per lo Studio e la Cura dei Tumori “Fondazione Giovanni Pascale” – IRCCS, Napoli, Italy; Gastrointestinal Surgery Unit, Istituto Nazionale per lo Studio e la Cura dei Tumori “Fondazione Giovanni Pascale” – IRCCS, Napoli, Italy; Medical Statistics Unit, Second University of Naples, Naples, Italy

**Keywords:** Rectal cancer, Short-course radiotherapy (SCRT), HDAC inhibitors, Valproic acid (VPA), FDG-PET, Preoperative chemo-radiotherapy

## Abstract

**Background:**

Locally advanced rectal cancer (LARC) is a heterogeneous group of tumors where a risk-adapted therapeutic strategy is needed. Short-course radiotherapy (SCRT) is a more convenient option for LARC patients than preoperative long-course RT plus capecitabine. Histone-deacetylase inhibitors (HDACi) have shown activity in combination with RT and chemotherapy in the treatment of solid tumors. Valproic acid (VPA) is an anti-epileptic drug with HDACi and anticancer activity. In preclinical studies, our group showed that the addition of HDACi, including VPA, to capecitabine produces synergistic antitumour effects by up-regulating thymidine phosphorylase (TP), the key enzyme converting capecitabine to 5-FU, and by downregulating thymidylate synthase (TS), the 5-FU target.

**Methods/Design:**

Two parallel phase-1 studies will assess the safety of preoperative SCRT (5 fractions each of 5 Gy, on days 1 to 5) combined with (a) capecitabine alone (increasing dose levels: 500–825 mg/m2/bid), on days 1–21, or (b) capecitabine as above plus VPA (oral daily day -14 to 21, with an intra-patient titration for a target serum level of 50–100 microg/ml) followed by surgery 8 weeks after the end of SCRT, in low-moderate risk RC patients. Also, a randomized phase-2 study will be performed to explore whether the addition of VPA and/or capecitabine to preoperative SCRT might increase pathologic complete tumor regression (TRG1) rate. A sample size of 86 patients (21-22/arm) was calculated under the hypothesis that the addition of capecitabine or VPA to SCRT can improve the TRG1 rate from 5% to 20%, with one-sided alpha = 0.10 and 80% power.

Several biomarkers will be evaluated comparing normal mucosa with tumor (TP, TS, VEGF, RAD51, XRCC1, Histones/proteins acetylation, HDAC isoforms) and on blood samples (polymorphisms of DPD, TS, XRCC1, GSTP1, RAD51 and XRCC3, circulating endothelial and progenitors cells; PBMCs-Histones/proteins acetylation). Tumor metabolism will be measured by 18FDG-PET at baseline and 15 days after the beginning of SCRT.

**Discussion:**

This project aims to improve the efficacy of preoperative treatment of LARC and to decrease the inconvenience and the cost of standard long-course RT. Correlative studies could identify both prognostic and predictive biomarkers and could add new insight in the mechanism of interaction between VPA, capecitabine and RT.

**EudraCT Number:** 2012-002831-28.

**Trial registration:**

ClinicalTrials.gov number, NCT01898104.

## Background

### Hystone deacetylases (HDAC) enzymes and role of HDAC inhibitors (HDACi) as anticancer agents

Histone deacetylases (HDACs) regulate the acetylation of a variety of histone and nonhistone proteins, controlling the transcription and regulation of genes involved in cell cycle control, proliferation, survival, DNA repair and differentiation. HDAC expression is frequently altered in hematologic and solid tumors [1].

Histone Deacetylase inhibitors (HDACi) represent a new class of antitumor agents able to affect, based on the function of the epigenetic enzymes they regulate, multiple genes and pathways [1–4]. In particular, our group and many others have demonstrated the synergistic antitumor activity of HDACi in combination with a large number of structurally diverse anticancer agents [2–5]. Many HDAC inhibitors (HDACi) have demonstrated preclinical efficacy as monotherapy or in combination with other anticancer drugs for both hematological and solid malignancies. However, clinical efficacy of HDACi, particularly in solid tumors, remains not demonstrated, most likely because of lack of understanding of the best context and combination regimen for their clinical use.

Several HDACi are currently in clinical development as anticancer agents and two (vorinostat and romidepsin) have been approved by the US FDA for the treatment of cutaneous T-cell lymphoma.

### Valproic acid: preclinical and clinical studies

The anti-epileptic valproic acid (2-propylpentanoic acid, VPA), an 8-carbon, branched-chained fatty acid, has HDAC inhibitory activity. Independent of this property, it is being used as an anticonvulsant agent and is clinically effective as a mood stabilizer in the treatment of maniac depression (bipolar affective disorder). The recommended values of serum concentrations for the treatment of epilepsy are in the 50–100 μg/ml range. Due to its HDAC inhibiting activity and its safe use as a chronic therapy (for over 40 years) for epileptic disorders, VPA has been considered a good candidate for anticancer therapy. In a large series of preclinical studies, exposure to VPA results in dose-dependent reversible cell cycle arrest and cell growth inhibition as well as chromatin decondensation and cellular differentiation in several neoplastic cell models [6].

Several phase I and II studies of VPA in adults with hematologic and solid malignancies showed that VPA treatment, either as a monotherapy or combined with other agents, was reasonably well tolerated and resulted in some encouraging tumor responses.

VPA ability to inhibit deacetylase activity in solid tumors has been demonstrated in monotherapy at oral doses between 20 and 60 mg/kg [7]. VPA oral doses of 30 mg/kg daily induced histone deacetylase inhibition in the peripheral blood of locally advanced breast cancer patients in a neoadjuvant therapy study in combination with the demethylating agent hydralazine added to doxorubicin and cyclophosphamide. The mean plasma concentration was of 87.5 μg/ml, the therapy was safe and tumor responses appeared higher as compared with historical controls [8].

In a phase I/II trial of VPA in combination with Epirubicin or in combination with 5-Fluorouracil, Epirubicin, and Cyclophosphamide (FEC100) for patients with solid tumors, 44 patients received escalating doses of valproate with a fixed dose of Epirubicin and the maximum tolerated dose (MTD) was 140 mg/kg/day with nine patients achieving a partial response. During the second part of the study, a disease-specific cohort of 15 breast cancer patients were treated with 120 mg/kg/day Valproate and the combination regimen FEC100. With nine out of 14 patients responding to therapy. Overall, somnolence was the most noted adverse effect related to VPA and the acetylation levels measured in peripheral blood mononuclear cell (PBMC) correlated with VPA serum levels and could be linked to baseline HDAC2-but not HDAC6 expression [9].

### VPA safety and cardiac toxicity

Common adverse effects associated with HDAC inhibitors include thrombocytopenia, neutropenia, diarrhea, nausea, vomiting and fatigue. Most toxicities are class-specific and have been observed with all HDAC inhibitors. However, differently from other HDAC inhibitors, VPA has a good safety profile with somnolence and neovestibular symptoms (dizziness, confusion) as dose limiting toxicities (DLTs), rather than fatigue [7–12].

A cardiac toxicity has been reported in several studies with other HDAC inhibitors [13–17]. In a phase I trial of VPA in combination with Epirubicin, a grade 2 QTc prolongation was reported in eight patients (18%), and a grade 3 QTc prolongation was seen in two patients (5%); these events occurred predominantly on day 1 of VPA treatment. QTc prolongations were associated with serum potassium levels less than 4.0 mmol/L and were resolved in all patients with appropriate potassium and magnesium supplementation [10].

### Rationale for the combination of an HDAC inhibitor with fluoropyrimidines and radiotherapy

Multiple HDAC inhibitors have been shown to affect radiosensitivity in preclinical models including VPA [18]. HDAC inhibitor vorinostat has been recently safely combined with short-term pelvic palliative radiotherapy in gastrointestinal neoplasms including rectal cancers [19]. A clinical trial combining VPA, radiation, and chemotherapy for children with high-grade gliomas reported that three times daily administrations, to maintain trough concentrations of 75 to 100 μg/ml of VPA, was well tolerated in children with refractory solid or central nervous system (CNS) tumors. Histone hyperacetylation in PBMCs was observed in half of the patients at steady state [20]. Moreover, a retrospective analysis of the dataset for the EORTC/NCIC chemo-radiotherapy trial with temozolamide and radiotherapy (RT) in newly diagnosed glioblastoma suggested that concomitant treatment with VPA might be associated with a prolonged survival [21].

*In vitro* and *in vivo* studies from our group and others, conducted in models of colon, head and neck and breast cancers, showed that treatment with HDACi is associated with the downregulation of thymidylate synthase (TS), the key enzyme in the mechanism of action of 5-Fluorouracil (5-FU) [5]. Moreover, we have recently demonstrated, for the first time, that HDACi vorinostat in combination with capecitabine produces a synergistic antitumor effects by up-regulating, in *vitro* and *in vivo*, in colorectal cancer cells but not in *ex vivo* treated peripheral blood lymphocytes, the mRNA and protein expression of thymidine phosphorylase (TP), the key enzyme converting capecitabine to 5-FU [2]. We confirmed a time and dose-dependent inhibition of TS and induction of TP mRNA and protein expression by several other HDACi, including VPA [2]. We investigated potential antitumor interaction between capecitabine metabolite 5′-deoxy-5-fluorouridine (5′-DFUR) and several HDACi showing synergistic/additive antiproliferative and proapoptotic effects in all cancer cells tested, with better results with VPA [22].

Interestingly, TP protein induction is achieved also at low doses of VPA (0.3-0.7 mM), corresponding to a plasma level between 50 and 100 μg/ml, easily reached in patients with normal anticonvulsant doses. Although at these doses VPA did not induce growth inhibition as single agents, a significant synergistic antitumor effect was still demonstrated in combination with 5′-DFUR, suggesting a specific mechanism of interaction [22]. TP knockdown experiments confirmed a crucial role of TP protein modulation in the observed synergism [2]. Moreover, washout experiments showed that the induction of TP, mediated by VPA treatment, is still evident 24 h after drug removal, suggesting the feasibility of a sequential-schedule of combination treatment [22].

### Definition of rectal cancer with low-moderate risk of recurrence

The shift from a postoperative to a preoperative chemo-radiotherapy (CRT) approach and the wide adoption of total mesorectal excision (TME) have remarkably improved the management of locally advanced rectal cancer (LARC), resulting in a significant improvement of local control [23]. Moreover, preoperative CRT, compared with postoperative CRT, significantly decreased acute and late toxicity, and increased preservation of sphincter function [23]. In the last years, because distant metastases have become the predominant pattern of failure in rectal cancer, the integration of new antineoplastic agents into preoperative fluoropyrimidine-based CRT has been studied. However, results from clinical trials, including randomized phase III trials, have showed disappointing results. Therefore, several novel strategies with different sequence of multimodal treatment components are being evaluated.

The evidence that LARC is a widely heterogeneous group of tumors with different prognostic behaviour [24], suggests that a risk-adapted therapeutic strategy should be pursued in this disease. Tumor (T) extension and lymph node (N) involvement represent important prognostic factors for recurrence-free and overall survival [25]. More recently, a prognostic role has also emerged for the circumferential resection margin (CRM) involvement that identifies patients with worse prognosis [26]. Moreover, the worse prognosis of patients with distal (less than 5 cm from the anal verge) rectal cancer has also been ascribed to the higher frequency of CRM involvement, occurring for the natural “coning-in” of the mesorectum in this location [27]. Currently, CRM involvement can be predicted by measuring the infiltration of perirectal fat from the mesorectal fascia (MRF) with high resolution magnetic resonance imaging (MRI); therefore, this test plays an important role in staging rectal cancer, because it may help to define patients prognosis. However, similarly to other imaging techniques, the accuracy of MRI in estimating lymph nodes involvements is limited. For this reason the management of clinical T3N0 is still controversial and preoperative RT or CRT is warranted for this subgroup of rectal cancer patients, despite the risk of overtreating early-stage disease [28].

Besides reducing local recurrence and improving survival, an additional goal in the treatment of rectal cancer is to perform conservative surgery, that can be safely indicated to patients with early T stage and node-negative cancer. However, the goal of sphincter preservation can be also pursued in more advanced cases, initially candidated to abdominal-perineal resection, thanks to preoperative RT [29].

Altogether, these findings suggest that, cT2N0 tumors located at <2 cm from anal verge, T2N1 or T3N0- N1 tumors, located at >5 cm from anal verge and with infiltration of perirectal fat >5 mm from MRF evaluated by MRI, can be categorized as a group of rectal cancer with low-moderate risk of recurrence, in which preoperative RT can be considered a valid option.

### Preoperative short-course radiotherapy

Radiotherapy has been extensively used in rectal cancer during the past decades to reduce the risk of a local failure, even if radical surgery seems feasible or has already been performed, or to increase the chances of a radical (R0) resection in a locally advanced tumour. In the first situation, a hypofractionated short-course radiotherapy (SCRT) followed with immediate surgery is an option supported by randomized trials, since no down-sizing or down-staging is required [30, 31]. In the second situation, conventionally fractionated long-course RT (1.8 Gy/fraction up to a final dose of 45 – 50.4 Gy) is used, followed by surgery 6 to 8 weeks later, to allow both the recovery from acute radiation-induced tissue reactions and tumor downstaging. Concomitant chemotherapy, 5-FU/capecitabine given along with the long-course RT improves local control [32–34] and it is thus a standard treatment for patients who are suitable for this combined therapy.

SCRT without chemotherapy has been compared with long-course CRT in two recent randomized studies and no statistically differences in recurrence rates and survival have been found [35, 36]. A Polish trial showed no difference in local recurrence rate and survival comparing conventional radiotherapy scheme (50.4 Gy, surgery after 4–6 weeks) combined with chemotherapy (5-FU/Leucovorin) with short-term preoperative radiotherapy (5 × 5 Gy, surgery within 7 days), although more down staging occurred with the former scheme [35]. Similar results were reported from a recent Australian trial [36]. An ongoing trial (Stockholm III) is randomizing patients with resectable rectal cancer to either long-course RT (50 Gy), SCRT with immediate surgery or SCRT with delayed surgery (4–8 weeks “waiting period”) and, recently, data from an interim analysis including 300 patients demonstrated that SCRT with delayed surgery is feasible [37]. Retrospective observational data have shown that SCRT with delayed surgery can produce significant downstaging and also pathological complete response (pCR) in some patients, with low toxicity [38–40]. In a trial including also M1 patients, systemic chemotherapy was administered after SCRT before surgery and no significant local tumor progression during chemotherapy was seen while in 11 of 41 resected rectal specimens a pathologic complete response was observed [41]. Altogether these data suggest that pre-operative SCRT with delayed surgery is feasible and that down-staging or down-sizing may occur following this regimen.

On this basis, and considering that SCRT is logistically convenient and cheaper when compared with CRT, it is interesting to assess the safety and efficacy of preoperative SCRT plus fluoropyrimidine-based chemotherapy followed by delayed surgery in patients with resectable rectal with low-moderate risk of recurrence.

### Rationale for the biologic pharmacogenetic and pharmacokinetic study

Histone acetylation in tumor samples and in PBMC correlated in several studies with VPA serum levels and were also further linked to baseline expression of some HDAC isoforms (i.e. HDAC2 but not HDAC6) [9, 42].

As mentioned above the synergism between HDACi and fluoropyrimidines seems explained by the modulation of the expression of TS and TP. Polymorphism of Dihydropyrimidine deydrogenase (DPD) gene or TS gene may affect toxicity and activity of fluoropyrimidines.

HDACi can regulate the expression of DNA repair genes such as RAD51 [43]. Polymorphisms in genes regulating DNA repair, such as XRCC1 (Arg399Gln), GSTP1 (lle105Val) RAD51 (135G > C) and XRCC3 (Thr241Met and 4541A > G), may affect activity and toxicity of radiotherapy.

Several reports demonstrated that circulating endothelial cells (CECs) levels are increased in the peripheral blood of cancer patients at diagnosis, and that chemotherapy can reduce the amounts of mature viable CECs determining the return to normal values in patients undergoing complete remission. In particular, Bertolini and colleagues have recently demonstrated that CEC count and viability could represent a promising predictive factor for anti-angiogenic therapies [44].

## Methods/Design

V-shoRT-R3 is a phase I/2 trial exploring the safety and the activity of capecitabine given alone or with VPA, during preoperative SCRT in patient with low-moderate risk rectal cancer.

### Objectives

The primary objective of the Phase I study is to determine the MTD of capecitabine given alone or in combination with valproic acid during preoperative SCRT.

The primary objective of the phase II comparative study is to explore whether the addition of valproic acid and/or capecitabine to SCRT before optimal radical surgery might increase the rate pathologic complete tumor regression (reported as tumor regression grade 1; TRG1) in patients with low-moderate risk rectal cancer.

Within each planned phase II comparison, secondary objectives include the evaluation of local control, disease free survival and overall survival, pathological CRM negative (>1 mm) and lymph node negative rate, short and long-term toxicity, surgical complications, and quality of life. The study also aims to validate the predictive role of early tumor metabolic changes measured by positron emission tomography (PET) scan (both phase I and II) and to assess the diagnostic accuracy of pre-surgical rectal biopsy, performed after the induction of anaesthesia in all surgical operations and analysed by intraoperative pathology (both phase I and II).

A translational sub-study is also planned, within the phase II trial, with several aims: (a) to compare the expression of several biomarkers (TP, TS, VEGF, RAD51, XRCC1, Histones and proteins acetylation, HDAC isoforms) in the tumor and normal mucosa, at baseline and at different time points during and after treatment; (b) to analyse polymorphisms of genes that may affect activity and toxicity of chemo-radiotherapy (DPD, TS, XRCC1, GSTP1, RAD51 and XRCC3) on DNA from peripheral blood; (c) to evaluate Circulating Endothelial Cells (CEC) and Progenitors (CEP) counts on peripheral blood at baseline and at different time points during and after treatment; (d) to evaluate Histones and proteins acetylation (H&P-Ac) of PBMCs at baseline and at different time points during and after treatment.

### Ethical aspects

The procedures set out in this study protocol are designed to ensure that the principles of the Good Clinical Practice guidelines of the International Conference on Harmonization (ICH) and the Declaration of Helsinki are respected in the conduct, evaluation and documentation of this study.

The study was approved by the Ethical Committee of the National Cancer Institute of Naples, Italy, and by the National Institute of Health, as required by the Italian regulation on Phase I clinical trials. Patients provide written informed consent for participating in the study and for allowing to collect tissue and blood samples.

### Study design

#### Phase I

**Figure 1:**
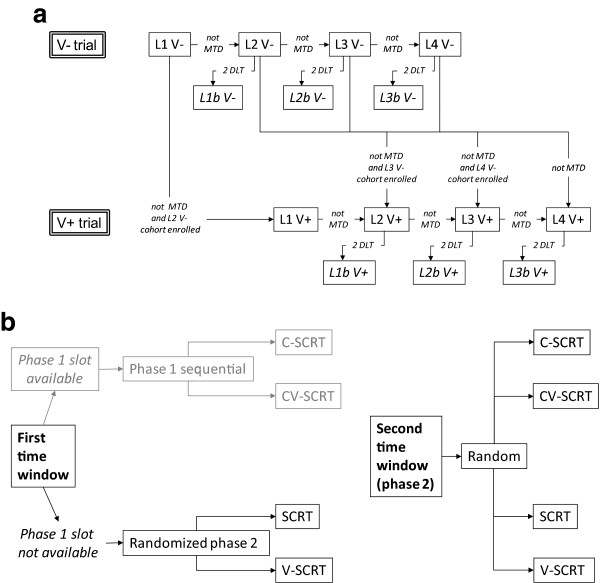
**V-shoRT-R3 study design. a)** Scheme of phase I study, **b)** Plan of Phase II study.

Two parallel phase I studies will be performed (Figure 1a) with capecitabine, given on days 1 to 21, concomitantly and after SCRT (5 fractions each of 5 Gy, on days 1 to 5), as single agent (V- trial) or in combination with VPA (V + trial) (orally daily from day -14 to 21, with an intra-patient titration for a target serum level of 50–100 μg/ml that is considered useful to produce the synergistic effect with radio- and/or chemotherapy).

Four increasing dose levels of capecitabine (L1-L4) are planned: 500, 650, 750, and 825 mg/m^2^/bid. Three further intermediate dose levels (L1b, L2b, L3b), are also provided in case MTD is reached (Figure 1a).

At each dose level, a cohort of 3 patients (pts) will be enrolled. Within each trial, MTD will be defined as the dose producing DLT in 2 pts (any grade 3 non-hematologic or grade 4 hematologic toxicity occurring within 5 weeks from day 1 of treatment).

Patients will be enrolled consecutively according to the available slot. However, the V + trial will begin after the enrollment of the first 2 cohorts in the V- trial. Subsequent cohorts in the V + trial will be enrolled only with dose levels of capecitabine that have not been defined as MTD in the V- trial.

The dose level immediately lower than the MTD will be the recommended phase II dose (RP2D); within each study, the cohort treated at the RP2D will be expanded up to 9 pts.

According to the study design, and if MTD is reached or not, sample size of each phase I study will vary from 2 to 33 pts.

Patients eligible at a time when phase I studies are still ongoing but a treatment slot is not available will be offered to enter the first time-window of the phase II trial (see below).

#### Phase II

The randomized phase II multicentre study will have two distinct time windows: the first one while phase I studies are ongoing and the second one after phase I studies have defined the RP2D of capecitabine (C) without and with VPA (V- and V+) (Figure 1b).

In the first window, randomization will be 1:1 to 2 arms, SCRT and V/SCRT, with patients randomized when a phase I slot is not available at the time of their inclusion. Its duration depends on the duration of phase I studies and there is no definite sample size.

In the second window, randomization will be 1:1:1:1 to 4 arms: SCRT; V/SCRT; C/SCRT CV/SCRT (Figure 1b).

The primary endpoint of the phase II study is the TRG1 rate according to Mandard modified scoring system [45], after definitive surgery.

We will conduct the randomized phase II study, with two separate comparisons, according to the following scheme:

- to test the effect of capecitabine: SCRT + V/SCRT vs C/SCRT + CV/SCRT;
- to test the effect of VPA: SCRT + C/SCRT vs V/SCRT + CV/SCRT.

Sample size for phase II study is calculated for the second time window. In details, a sample size of 86 patients (approximately 21–22 pts assigned to each arm) is planned under the hypothesis that the addition of capecitabine or VPA to SCRT can improve the TRG1 rate from 5% to 20%, with one-sided alpha = 0.10 (that is 0.20 corrected for the 2 planned comparisons) and 80% power. Patients randomized during the first time window, will be added to the analysis of the effect of valproic acid; as a consequence, the statistical power of such comparison will be increased and more reliable estimates of treatment toxicity will be produced. Randomization will be performed with a minimization procedure that will account for centre, clinical N stage (N0 vs N1) and clinical T stage (T2 vs T3).

### Patient selection criteria

#### Inclusion criteria

Patients ≥18 and ≤70 years, diagnosed with adenocarcinoma of rectum defined at low-moderate risk of recurrence by T and N extension but also on the basis of CRM involvement measured by MRI: cT2N0 tumors located at ≤2 cm from anal verge, T2N1 or T3N0- N1 tumors, located at >5 cm from anal verge and with infiltration of perirectal fat >5 mm from MRF evaluated by MRI. ECOG Performance Status ≤1. Effective contraception for both male and female patients if the risk of conception exist. Signed written informed consent.

#### Exclusion criteria

Any previous pelvic radiotherapy or treatment for rectal cancer. Presence of metastatic disease or recurrent rectal tumor. History of inflammatory bowel disease or active disease. Any concurrent malignancy. Inadequate bone marrow, liver or renal function (Neutrophils <2000/mm3 or platelets <100.000/ mm3 or haemoglobin <9 gr/dl; Creatinine levels indicating renal clearance of <50 ml/min; GOT and/or GPT >2.5 time the upper-normal limits, UNL; and/or bilirubin >1.5 time UNL). Significant cardiovascular comorbidity. Patients with long QT-syndrome or QTc interval duration >480 msec or concomitant medication with drugs prolonging QTc. Patients who cannot take oral medication.

Patient who have had prior treatment with an HDACi and patients who have received compounds with HDACi-like activity, such as valproic acid. Known or suspected hypersensitivity to any of the study drugs. Concurrent uncontrolled medical conditions that might contraindicate study drugs. Major surgical procedure, within 28 days prior to study treatment start. Pregnant or lactating women.

### Treatment plan

**Figure 2:**
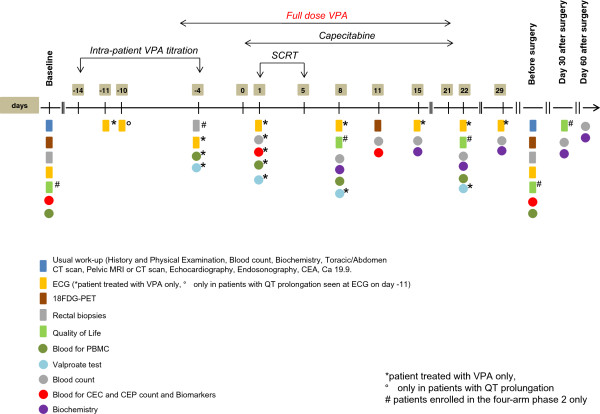
**Schematic timeline of study procedures.** Note. History and physical examination, blood count, biochemistry will be repeated weekly during treatment.

Treatment with VPA will not be matter of dose-finding but a titration strategy will be applied in each patient looking for a serum concentration that is considered useful to produce the desired synergistic effect with radiotherapy and/or chemotherapy. Treatment will be administered orally starting at day -14, until day 21 from beginning of radiotherapy, with a 500 mg slow releasing tablet at evening (Figure 2). Thereafter, the dose will be increased also using 300 mg tablets (Table 1). In the morning of day -4, serum level of VPA will be checked and will be adjusted depending on the reached steady level. The target serum level range will be 50–100 μg/ml which represents the recommended values for the treatment of epilepsy. At any time, in case of grade 2 somnolence or fatigue the VPA dose will be reduced by 200 mg/day steps up to reaching grade ≤1 independently of the actual serum level. In case of grade ≥3 somnolence or fatigue VPA will be definitely suspended. In case of asymptomatic QTc prolongation development (QTc >500 ms, or QT prolongation >600 ms,) VPA has to be suspended. Electrolytes and concomitant medications have to be checked and corrected. ECG has to be repeated after 24 hours. If the event is resolved, treatment with VPA can be resumed but the dose will be reduced by -200 mg/day; on the contrary, if QT prolongation is confirmed VPA has to be interrupted [46, 47] .

**Table 1:** **Valproic acid titration scheme**

Days	Morning dose*	Midday dose*	Evening dose*
-14 & -13	0	0	500
-12 & -11	300	0	500
-10 & -9	500	0	500
-8 & -7	500	300	500
-6 & -5	500	500	500
-4 & -3	500	500	500
-2 & -1	500	500	500

*The interval between doses will be 12 hours on days -14 to -9 and 8 hours from day -8.

In case of symptomatic QTc prolongation development (QTc >500 ms or QT prolongation >600 ms associated with symptoms suggestive of a ventricular tachyarrhythmia), VPA has to be interrupted.

During phase I studies, capecitabine will be administered according to the dose-finding scheme reported above, therefore at a daily dose ranging from 500 mg/m2/bid to 825 mg/m2/bid, for 21 days starting on the day before the beginning of radiotherapy. During the phase II study, capecitabine will be administered at the daily dose indicated as RP2D within phase I studies. This dose might be different without and with VPA, according to results of phase I studies. Toxicity due to capecitabine will be managed by 25% dose-reduction in case of grade 2 adverse events. Treatment with capecitabine will be interrupted upon the occurrence of a grade ≥3 adverse event and restarted once the adverse event has resolved or decreased in intensity to grade 1. The dose will be maintained after the 1st occurrence of the event and will be reduced by 25% at each subsequent occurrence up to a maximum of 50%. Treatment should be discontinued after the 4th occurrence of the event.

RT will be administered according to a short course hypofractionated scheme (SCRT) consisting of five fractions each of 5 Gy for 5 consecutive days for a total dose of 25 Gy.

Both in phase I and II studies, surgical operation, will be performed 8 weeks after the last day of radiotherapy. To explore diagnostic accuracy of pre-surgical rectal biopsy, all surgical operations will include a preliminary rectal biopsy, that will be performed after the induction of anaesthesia. The biopsy specimen will be examined intraoperatively; however, the result of intraoperative pathology will not influence subsequent surgical behaviour. An anterior resection or an abdominal perineal resection, with total mesorectal excision, will be performed on the basis of restaging. Fecal diversion to protect the anastomosis will be performed by the means of a loop ileostomy; and ileostomy reversal will be performed after endoscopic assessment of anastomotic integrity. All resection specimens will be examined by two independent dedicated rectal cancer pathologists and pathologic staging, ypTNM and TRG, will be determined according to AJCC guidelines [48]. The number of examined/involved lymph nodes, tumor differentiation, lymphatic and venous invasion, and status of proximal, distal, and circumferential resection margins will be also reported.

### Assessment and procedures

Assessment and procedures, including those for exploratory objectives (see below), are illustrated in Figure 2.

### Adverse events

Adverse events will be graded according to the Common Terminology Criteria for Adverse events of the National Cancer Institute (CTCAE-NCI) version 4.0.

Adverse events will be assessed at the following times: at baseline (within 3 weeks before the initiation of any treatment), at days 8, 15, 22, 29, before surgery, 1 and 2 months after surgery. In addition, only in patients receiving VPA, adverse events will be assessed at day -4.

### FDG-Positron Emission Tomography (PET) imaging

MRI and other conventional imaging modalities such as EUS and CT are unable to differentiate post-radiation inflammation and fibrotic changes from viable tumor in the residual lesion following preoperative treatment [49, 50]. In contrast, metabolic imaging with [18 F] 2-fluoro-2-deoxy-D-glucose positron emission tomography (FDG-PET) may be more valuable in this respect as the high glycolitic activity of tumor cells can be utilized to discriminate fibrosis from viable tumor tissue [51]. In the neoadjuvant setting, a strong correlation between FDG standardized uptake value (SUV) changes and pathologic response has been demonstrated in different tumors [52–54], including rectal cancer [55]. Our group has previously reported that early metabolic change evaluated by FDG-PET is able to predict pathologic tumor response [56] and outcome [57] in rectal cancer. Thus, FDG-PET/CT scans are planned at baseline and on day 11 (+/- 2) in patients enrolled in phase II study, to validate the ability of early metabolic change to predict TRG and outcome. For each tumor volume, maximal standardized uptake value (SUV-max, the maximum pixel value in the lesion), SUV-mean (the average SUV value in the lesion) and Total Lesion Glycolisis (TLG, SUV-mean x metabolic tumor volume) will be calculated. A responder patient, consistent with our previous studies, will be define according to reduction of SUV or TLG parameters of 50% or more compared to baseline. Therefore, patients with any change below this threshold will be defined as non-responder. Further thresholds will be eventually explored only in case of failure (lack of predictive ability) of the proposed validation.

### Pharmacodynamic, pharmacogenetic and pharmacokinetic studies on tumor and blood samples

Tumor and normal mucosa samples will be collected only in patients enrolled in the four-arm phase II study: at baseline (possibly within the diagnostic rectal biopsy) and at surgery for all patients and at day -4 in patients assigned VPA.

Baseline tumor expression of TP, TS, VEGF, RAD51 and XRCC1, will be compared with normal mucosa and with tumor expression at the following time points as pharmacodynamic/predictive markers of treatments (analyzed by real-time PCR and immunohystochemistry). In fact, as reported above, several evidences, by our group and others, including preliminary results, suggested a crucial role of the modulation of the expression of TS and TP in the synergism observed between HDACi and fluoropyrimidine. On the other hand, TP showed a strong sequence homology to the pro-angiogenic platelet derived endothelial cell growth factor (PD-ECGF), and may contribute to angiogenesis, tumor progression and metastasis. However, several reports have clearly shown that HDACi inhibit tumor-induced angiogenesis by regulating VEGF production and signaling. Moreover, the expression DNA repair genes, such as RAD51 or XRCC1, affecting sensitivity to RT and or chemotherapeutics, can be also regulated by HDACi.

Histones and proteins acetylation (H&P-Ac) measured at all the time points and HDAC isoforms evaluated at baseline represent additional pharmacodynamic/predictive specific markers of VPA HDACi activity.

Peripheral blood samples will be collected at baseline, on day -4, 1, 8, 11, 22 and at surgery. VPA serum level will be measured by a valproate test at all time points and correlated with H&P-Ac, measured on peripheral PBMC as additional surrogate pharmacodynamic markers of VPA activity by multiparametric flowcytometry.

Polymorphisms of genes that may affect activity and toxicity of radio-chemotherapy such as DPD, TS, XRCC1, GSTP1, RAD51 and XRCC3, will be analyzed on baseline samples by pyrosequencing technology.

Circulating Endothelial Cells (CEC) and Progenitors (CEP) counts will be analyzed as surrogate marker of tumor angiogenesis at baseline, on day 1, 11 and at surgery by multiparametric flowcytometry.

### Quality of life assessment

Quality of Life (QoL) will be assessed in patients enrolled in the randomized four arm phase II study by the EORTC QLQ-C30, version 3.0, and the EORTC QLQ-CR29 questionnaires that will be filled in by patients before treatment, at the end of radiotherapy (D8) and of chemotherapy (D22), before surgery and 30 days after surgery [58, 59].

### Adjuvant treatment

There is no general agreement on the benefit of adjuvant CT after preoperative CRT. The only study, EORTC trial 22921, to formally evaluate the benefit of adjuvant CT after preoperative CRT failed to demonstrate a significant impact on survival of postoperative chemotherapy [33]. Moreover, emerging data suggest a significant correlation between pathologic response to preoperative chemo-radiotherapy and oncologic outcomes, evidencing the favorable prognostic value of pathologic complete response [60–62]. These data generate the hypothesis that it might be reasonable to link the decision on adjuvant treatment to the pathologic response obtained after neoadjuvant treatment. However, given the absence of definitive evidence on this topic, and considering that the impact of adjuvant treatment in this protocol will only affect secondary end-points, decision regarding adjuvant chemotherapy will be decided by the investigators according to the policy commonly adopted by their Institution in clinical practice.

### Follow up

Patients will have follow-up evaluation every three months for three years and every six months during the following two years. Patients who have discontinued study treatment for reasons other than progressive disease will enter follow-up.

### Statistical analysis of phase II

Phase II analysis will be performed according to the intention-to-treat strategy. Analyses will be performed separately for the two planned comparisons. In each comparison, TRG1 rate is defined as the rate of patients out of those randomized who will experience a complete pathological regression according to Mandard modified scoring system (responders). Patients who will not achieve a TRG1 will be defined as non-responders. Patients who will not undergo primary surgery because of progressive disease will be defined as non-responders. TRG1 rates will be compared with chi-square test in a 2×2 contingency table (responders/non-responders x treatment arms).

For each patient and for each type of toxicity, the worst degree suffered during treatment will be used for the analysis. Two sets of statistical analyses will be performed to compare toxicity. In the first set the whole pattern of toxicity (all grades) will be considered for each item; analysis will be done by a linear rank test. In the second set toxicity will be defined as severe (mostly including grade 3 or higher) and not severe (mostly including grades up to 2) and analysis will be performed by Fisher’s exact test.

Due to the small sample size, statistical analysis of biomarkers data will be conducted with the aim of hypothesis generation. Biomarkers that might change over time as a consequence of treatment, levels before and after treatment will be compared with appropriate statistical tests, based on the type of data.

QoL will be described according to EORTC rules [58, 59].

## Discussion

The goal of the study is to demonstrate the feasibility and explore the activity of a preoperative treatment with SCRT, a very convenient modality of RT, in combination with capecitabine and/or VPA, in patients with low-moderate risk rectal cancer, and to identify potential biomarkers predictive of toxicity and efficacy for these combinations.

To date, the optimal preoperative management of RC remains controversial regarding RT fractionation, timing of surgery and use of concurrent chemotherapy.

Although preoperative SCRT has been assumed as a valid option in resectable RC, with similar outcome but low acute toxicity compared to long-course chemo-RT, only one recent study investigate the feasibility of SCRT plus 5FU [63]. Thus, our study is the first to investigate both feasibility and activity of SCRT plus Cap. We will also test for the first time the addition of VPA, a safe and low cost generic drug with HDACi activity, to SCRT ± Cap.

This approach might improve the efficacy of preoperative treatment of LARC and decrease its inconvenience and cost as compared to the standard long-course chemo-radiotherapy.

We will also evaluate mechanistically-based pharmacokinetic/pharmacodynamic biomarkers on tumor and blood samples and the predictive role of early (within 11 days after the beginning of SCRT) tumor metabolic changes measured by 18FDG-PET/TC in patients undergoing phase II trial. These correlative studies could identify both prognostic and predictive biomarkers and could add new insight in the mechanism of interaction between VPA, capecitabine and RT. Furthermore, the identification of biomarkers predictive of pathologic tumor regression, including early tumor metabolic changes measured by 18FDG-PET/TC, could improve the selection of patients candidate to a conservative surgical approach, which represent a major goal, considering the morbidity of total mesorectal excision and its impact on patients quality of life and costs.

### Trial sponsorship

The study is a non-profit academic investigator initiated trial promoted by Istituto Nazionale Tumori di Napoli G. Pascale who will provide insurance policy and drugs. The study is partially supported by grants to AB from Italian Association for Cancer Research and Italian Ministry of Health that play no role in protocol definition, trial performance and data analysis and interpretation.

## Abbreviations

(18FDG-PET): [18 F]2-fluoro-2-deoxy-D-glucose positron emission tomography
(5′-DFUR): 5′-deoxy-5-fluorouridine
(5-FU): 5-Fluorouracil
(C): Capecitabine
(CNS): Central nervous system
(CRT): Chemo-radiotherapy
(CECs): Circulating endothelial cells
(CEP): Circulating endothelial progenitors cells
(CRM): Circumferential resection margin
(CT): Computed tomography
(DPD): Dihydropyrimidine dehydrogenase
(DLT): Dose limiting toxicity
(EUS): Endorectal ultrasound
(HDAC): Histone deacetylase
(HDACi): Histone-deacetylase inhibitors
(H&P-Ac): Histones and proteins acetylation
(LARC): Locally advanced rectal cancer
(MRI): Magnetic resonance imaging
(MTD): Maximum tolerated dose
(MRF): Mesorectal fascia
(TRG1): Pathologic complete tumor regression
(pCR): Pathological complete response
(PBMC): Peripheral blood mononuclear cell
(PD-ECGF): Platelet derived endothelial cell growth factor
(PET): Positron emission tomography
(QoL): Quality-of-life
(RT): Radiotherapy
(RP2D): Recommended phase II dose
(SCRT): Short-course radiotherapy
(SUV): Standardized uptake value
(TP): Thymidine phosphorylase
(TS): Thymidylate synthase
(TLG): Total lesion glycolisis
(TME): Total mesorectal excision
(VPA or V): Valproic acid.
